# Future-proofing agricultural research: FAIR principles for agriculture AI agents (FAIR4AG^2^)

**DOI:** 10.3389/frai.2026.1877344

**Published:** 2026-08-06

**Authors:** Chenhao Qian, YeonJin Jung, Haowen Hu, Yijing Gong, Ariana Nicole Negreiro, Victor E. Cabrera, Renata Ivanek, Martin Wiedmann

**Affiliations:** 1 Department of Food Science, Cornell University, Ithaca, NY, United States; 2 Department of Animal Science, Cornell University, Ithaca, NY, United States; 3 Department of Animal & Dairy Sciences, University of Wisconsin-Madison, Madison, WI, United States; 4 Department of Population Medicine and Diagnostic Sciences, Cornell University, Ithaca, NY, United States

**Keywords:** agentic AI, agricultural research, FAIR (findable accessible interoperable and reusable) principles, large language model (LLM), research data infrastructure

## Abstract

The rapid shift of large language models from conversational use to agentic reasoning is changing how scientific outputs must be structured for machine consumption. Agriculture stands to gain the most from this transition but currently has the least of the centralized, machine-ready infrastructure that biomedicine has built over decades. Agricultural knowledge remains dispersed across peer-reviewed journals, extension bulletins, technical reports, and multimedia field demonstrations, with associated code, data, and models often inaccessible to autonomous agents. We argue that the foundational FAIR principles and FAIR for Research Software (FAIR4RS) must be extended to a new standard of agent-actionability, and propose FAIR4AG^2^ as that extension for agricultural research. Across three modalities of knowledge units (text, multimedia, and databases), we offer concrete, implementation-ready practices: structured publishing formats and machine-readable licensing for documents; signal isolation, time-aligned visuals, and domain-aware curation for multimedia; and standardized APIs, Agent Skills, and Model Context Protocol (MCP) servers for databases and model repositories. Realizing FAIR4AG^2^ will require parallel investment in equitable participation, careful curation, human-in-the-loop verification, and governance norms that credit the data curators and infrastructure builders whose work agents now operate upon.

## Introduction

The rapid evolution of frontier large language models (LLMs) is catalyzing a shift of artificial intelligence (AI) tools from conversational use to agentic reasoning and task completion. In this new paradigm, AI systems act as agents capable of autonomously formulating multi-step plans and executing actions within complex digital environments to solve research problems. This potential is already being realized through rapid progress in scientific agents such as the Virtual Lab (Swanson et al., 2025), which coordinates specialist agents to design nanobody candidates for SARS-CoV-2.

**Figure 1:**
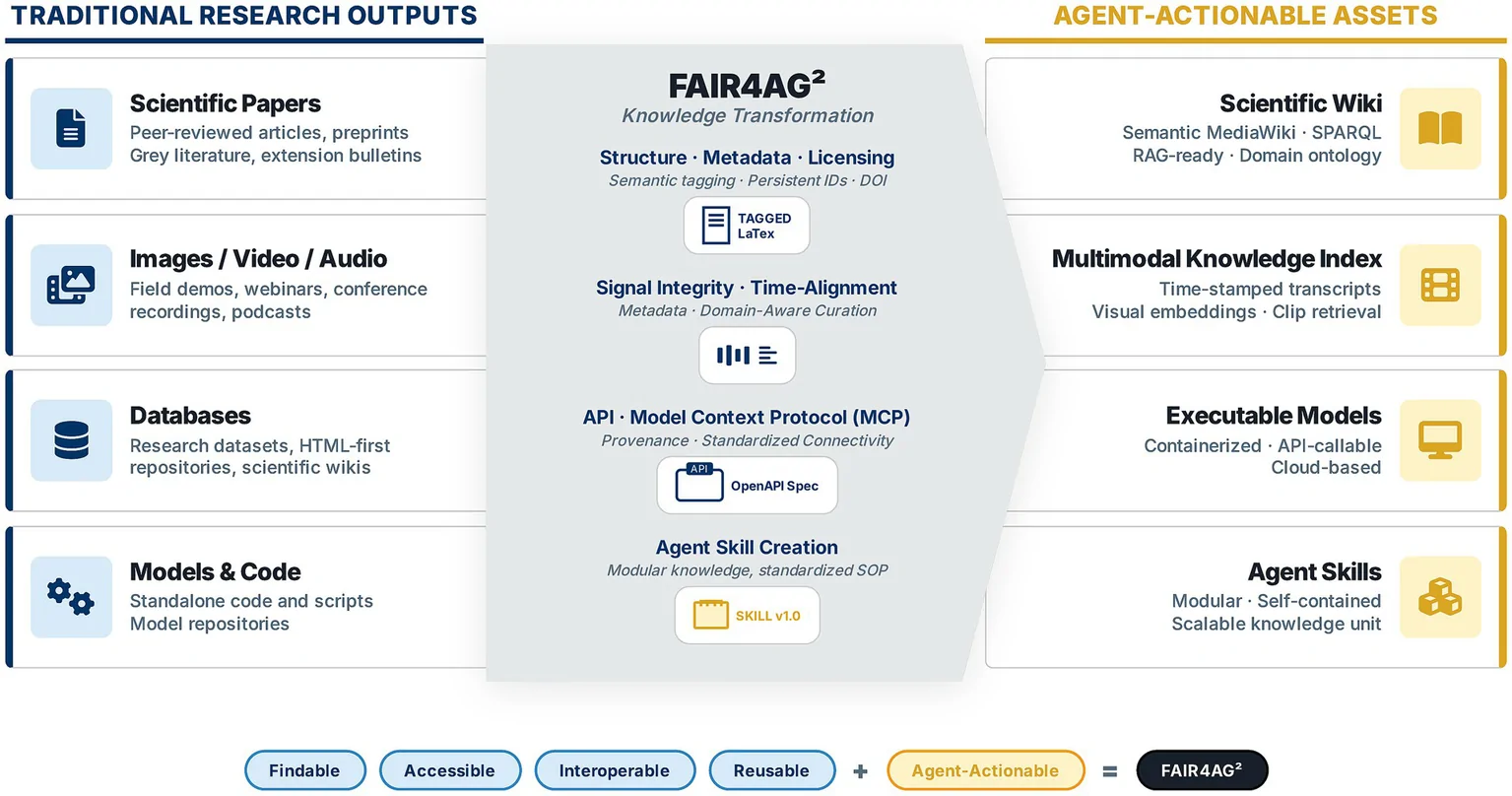
Illustration for applying FAIR4AG^2^ principles.

This trajectory is now extending into agricultural domains, fostering specialized systems for decision support. Applications range from high-throughput phenotyping in plant science (Awais et al., 2025) to complex decision-making frameworks in dairy science (Cabrera et al., 2025; Gontijo et al., 2025; Liu et al., 2025). Parallel work in crop protection demonstrates that knowledge-graph-grounded question answering raises pest and disease Q&A accuracy from 72.6% with naive RAG to 89.4% (Wu et al., 2026). By leveraging techniques such as tool calling (which enables agents to directly query databases or execute decision-support models) and Retrieval-Augmented Generation (RAG) from a curated knowledge base, AI agents can synthesize domain evidence to provide operational guidance. However, many agricultural research outputs remain “black boxes”; for many papers, the underlying data, code and models are not accessible (e.g., via repositories or containerized services), creating barriers for agents to execute, or build upon published content. Addressing this bottleneck requires us to revisit and extend the foundational FAIR (Findable, Accessible, Interoperable, and Reusable) data principles (Wilkinson et al., 2016) and FAIR for Research Software (FAIR4RS) (Barker et al., 2022). While these two frameworks successfully established standards for human-centric findability and reproducibility, the era of agentic AI demands a new standard: Agent-actionability. We define agent-actionability as the degree to which a knowledge unit can be discovered, accessed/authenticated, executed, provenance-traced, and its outputs interpreted by an autonomous agent without human intervention. We provide recommendations below on knowledge units (Table 1) with measurable criteria, and compare FAIR4AG^2^ against FAIR and FAIR4RS to show what each principle adds to previously established principles to address agent-actionability gap (Table 2). Agriculture is uniquely positioned to benefit from, and uniquely unprepared for, this transition. Unlike the biomedical sciences, which have invested decades in centralized infrastructure (PubMed Central, NCBI, ENA) and now host AI-native tooling such as Paperclip (n.d.) that lets researchers query and reason over millions of indexed papers from the command line, agricultural knowledge remains dispersed across heterogeneous sources: peer-reviewed journals, extension bulletins, government technical reports, farm or facility records, and multimedia field demonstrations. This heterogeneity, combined with the discipline’s heavy reliance on grey literature and its tight coupling to operational, time-sensitive decisions, means agriculture stands to gain the most from agent-actionable infrastructure while currently having the least of it (Top et al., 2022). FAIR4AG^2^ is therefore not a generic restatement of FAIR for a different community, but a response to a structural gap (see Figure 1).

**Table 1:** Proposed FAIR4AG^2^ principles with agent-actionability maturity anchors.

Principle		Unmet (0)	Partial (1)	Met (2)
To be findable				
F1	Knowledge units are assigned a globally unique and persistent identifier, with version- and component-level identifiers.	No persistent identifier; located only via a mutable URL.	Persistent identifier present, but no version- or component-level identifiers.	Persistent identifier resolvable at version and component level.
F2	Knowledge units are described with rich, machine-readable metadata.	No structured metadata; free text or none.	Metadata present but incomplete or human-facing only (not machine-parseable).	Rich metadata in a machine-readable schema.
F3	Metadata use domain ontologies and URIs to unambiguously tag and link concepts across resources.	Concepts in free-text terms only; no ontology linking.	Keyword tagging present but not anchored to ontology URIs/inconsistent vocabulary.	Concepts tagged and linked via domain-ontology URIs.
F4	Knowledge units are registered or indexed in searchable registries to support programmatic discovery.	Not indexed; discoverable only by prior knowledge of its location.	Listed in a human-browsable catalog, but not programmatically queryable.	Indexed in a registry supporting programmatic discovery.
To be accessible				
A1	Knowledge units are retrievable by identifier via standardized, open, universally implementable protocols.	Retrieval requires manual or bespoke steps; no standard protocol.	Retrievable via a protocol, but proprietary or not resolvable from the identifier alone.	Retrievable by identifier over standardized, open protocols.
A2	Where necessary, protocols support authentication/authorization suitable for agents.	Access blocked to agents where auth is needed (e.g., interactive login/CAPTCHA only).	Authentication exists but not agent-compatible (no token/key-based flow).	Standardized, agent-compatible auth where required.
A3	Models and agent skills/tools are accessible through execution interfaces (e.g., APIs, MCPs and/or containerized services), not only as downloads.	Available only as a download / static file.	Executable by humans via browser UI, but no programmatic execution endpoint.	Exposed via API/MCP/containerized service that agents can invoke.
A4	Metadata remain accessible even when the underlying asset is no longer available.	Metadata disappear with the asset (dead link leads to nothing).	Some metadata retained but not guaranteed (no tombstone record).	Metadata remain resolvable (e.g., tombstone) after asset removal.
To be interoperable				
I1	Knowledge units use formal, accessible, shared languages for representing inputs/outputs, schemas, and metadata.	Inputs/outputs/schemas in *ad hoc* or undocumented formats.	Common formats used, but no formal/shared schema definition.	Formal, documented, shared languages/schemas for I/O and metadata.
I2	Knowledge units include qualified, typed references to other objects.	References are inline text/hyperlinks, untyped, no identifiers.	References use identifiers but relationships are untyped/unqualified.	Typed, qualified references using persistent identifiers.
I3	Provenance is version-controlled and machine-readable, enabling end-to-end traceability of resources used and transformations applied.	No provenance or lineage recorded.	Provenance documented for humans but not version-controlled/machine-readable (e.g., coarse wiki edit history).	Version-controlled, machine-readable provenance enabling end-to-end traceability.
I4	Multimodal resources include alignment metadata (e.g., time-aligned slides/transcripts) to link claims to evidence.	Multimodal content with no cross-modal alignment.	Transcript or slides present, but not time-aligned/linked.	Time-aligned metadata linking claims to visual/audio evidence.
To be reusable				
R1	Knowledge units are richly described with accurate attributes, including applicability conditions and limitations.	Minimal or no description of applicability/limitations.	Descriptive attributes present but omit applicability conditions or limitations.	Rich attributes with explicit applicability conditions and limitations.
R2	Knowledge units are released with clear, machine-readable licenses covering text, code, and data.	No license, or stated only in unstructured prose.	License present but not machine-readable, or ambiguous across versions (e.g., AAM vs. VoR).	Machine-readable, per-version license covering text, code, and data.
R3	Agent skills/tools emit machine-readable runtime records (parameters, versions, environment) to support reproducibility and auditing.	No runtime records emitted.	Some logging, but not machine-readable or missing versions/environment.	Machine-readable runtime records (parameters, versions, environment).
R4	Knowledge units meet domain-relevant community standards.	Does not conform to recognized community standards.	Conforms to general standards but not domain-specific ones.	Conforms to relevant domain standards (e.g., FSKX for food-safety models).

Each principle is assessed on a three-level scale—unmet (0), partial (1), met (2)—where “partial” denotes a state that serves human users but falls short of autonomous agent use.

**Table 2:** Comparative analysis of FAIR, FAIR4RS, and FAIR4AG^2^ across the four principle categories.

Principle	FAIR (Wilkinson et al., 2016)	FAIR4RS (Barker et al., 2022)	FAIR4AG^2^
Findable	Data are assigned with persistent identifiers and rich metadata, and indexed in searchable resources	Software is assigned with persistent identifiers with version-level identifiers. Metadata are searchable and indexable	Knowledge units (data, models, agent skills/tools) are assigned with version and component-level identifiers and indexed in registries that are searchable by agents
Accessible	Data are retrievable by identifier via standardized, open protocols. Metadata still persists when data are not	Software is retrievable via open protocols, accounting for software’s dependencies	Knowledge units are retrievable via standardized protocols and execution interfaces are added so that the models and skills can be called by agents
Interoperable	Data use formal, shared languages and includes qualified references to other data	Software interoperates via standard formats and qualified references to other objects	Knowledge units use formal, shared languages and adds machine-readable, version-controlled provenance for end-to-end agent traceability, and align metadata for multimodal resources
Reusable	Data are richly described with clear license and meet community standards	Software is released with clear license, associated with detailed provenance and follows community standards	Knowledge units are richly described with accurate attributes and adds runtime provenance logging and machine-readable licensing

Concretely, we argue that Findability must evolve from simply searching for keywords to enabling agents to discover specific skills and tools they can use autonomously. Accessibility must move beyond downloadable files to standardized interfaces that allow agents to retrieve data without human assistance. Interoperability prioritizes clear version control and metadata for every knowledge unit and tool, enabling an agent to display an explicit chain of thought to trace which resources were used to make a decision. Finally, Reusability must ensure that research software is not just documented for a reader, but built as modular, executable tools that an agent can pick up and apply to new problems. In the sections that follow, we translate these principles into concrete, implementation-ready practices for modalities, such as text, multimedia, and databases, to make agricultural research more agent-actionable without imposing unrealistic burdens.

## Text based documents

Text-based documents, ranging from peer-reviewed journal articles to “grey literature” (e.g., technical reports, extension bulletins), remain the primary vehicle for agricultural knowledge. Although frontier LLMs are increasingly capable of ingesting and summarizing text across diverse formats and layouts, this progress does not eliminate a persistent barrier: without consistent structure, explicit metadata, and stable links to associated datasets, agents cannot reliably retrieve and interpret research outputs at scale. We therefore recommend a set of practical conventions to make agricultural documents more agent-actionable.

### Structure

The structural format of the document dictates its utility for AI agents. For academic research articles, we advocate for the use of structured formats like LaTeX, which preserves the semantic hierarchy of data, equations, and tables better than PDFs. Platforms such as Overleaf (AI features—Overleaf, Online LaTeX Editor, n.d.) and OpenAI PRISM (Prism|A free, LaTeX-native workspace for scientists|OpenAI, n.d.) have been instrumental in democratizing LaTeX by offering an intuitive, interactive interface that lowers the technical barrier for researchers. Publishers should support this by providing validated Overleaf templates. For Extension materials and technical reports where standard word processors remain the norm, we recognize the technical challenges of writing in LaTex format. In these cases, the focus must shift to post-publication processing; publishers and institutions who intend to make their publications more accessible could invest in emerging AI-assisted OCR tools [e.g., Landing AI (LandingAI: Computer Vision Platform—Visual AI Software, n.d.) or OLMoCR (olmOCR—Open-Source OCR for Accurate Document Conversion, n.d.)] that can reliably extract structured data from “messy” legacy formats.

### Metadata and licensing

High-quality metadata transforms agricultural research from searchable to actionable by autonomous agents (Table 1). While modern AI agents are increasingly proficient at inferring context, textual ambiguity forces models to guess connections rather than deterministically link concepts via concept Uniform Resource Identifiers (URIs). The need for domain specific ontologies is particularly emphasized in the agri-food sector (Top et al., 2022). To ensure accuracy, we recommend augmenting submissions with semantic tags, prioritizing resources with transparent, agent-ready infrastructure. FoodOn (n.d.) and AGROVOC (n.d.) exemplify this standard. Hosted on development platforms like GitHub, FoodOn offers transparent version control, allowing agents to trace exactly which definition was used. Similarly, AGROVOC provides a robust SPARQL endpoint, enabling agents to dynamically query concept relationships without downloading massive datasets. AgroPortal (Jonquet et al., 2018) complements these by serving as a centralized API to discover and align these vocabularies.

A critical but often overlooked component is clear licensing information, particularly given the complexity of article versioning. A single publication typically exists in multiple forms: the Author Accepted Manuscript (AAM), which is the peer-reviewed content prior to publisher typesetting, and the Version of Record (VoR), the final published article. These versions often carry different rights to reuse. For a human reader, distinguishing these versions is trivial. However, for an AI agent, ambiguity regarding which version it has accessed is a critical failure mode. If a license is not explicitly encoded in the metadata (e.g., via Schema.org or Dublin Core), compliant AI systems may exclude such content to avoid legal liability, which is a practice reflected in developers who, facing pervasive licensing ambiguity (Longpre et al., 2024), train only on permissively licensed data (Kocetkov et al., 2022). Therefore, we recommend that publishers include a concise, machine-readable licensing table specifying rights for text mining and reuse for each version, to provide the legal certainty and operational clarity for AI agents.

### Figures, data, and citations

Current publishing practices often sever the connection between a figure and its underlying analysis. Since modern visualizations result from complex data processing, we recommend linking figures to their generative code by explicitly referencing the specific script (e.g., fig 1_plot.R) in the caption. This connection allows an AI agent to re-execute the pipeline, transforming the figure from a static image into a verifiable asset. We also recommend formally referencing datasets and software in the bibliography using persistent identifiers (PIDs) rather than inline hyperlinks. This enables AI tools to traverse the citation graph, automatically linking a paper to the precise version of the model or dataset used to generate its conclusions.

## Images, audio and videos

Audio and video materials (e.g., podcasts, webinars, conference recordings, field demonstrations) are essential components of scientific communication in agriculture. These formats are especially valuable for building domain-specific knowledge bases that support Large Multimodal Models (LMMs) in advancing science and education. However, challenges persist in standardizing this data for AI ingestion. Without intentional standardization, much of this content remains difficult for AI systems to ingest, interpret, and reuse. Below we highlight three priority areas where targeted improvements can directly increase the scientific and computational value of agricultural multimedia.

### Signal integrity

Agricultural multimedia is generated across diverse platforms, introducing variability in quality and machine compatibility (Kpodo and Nejadhashemi, 2025). Suboptimal recording conditions (such as audio-video lags in conference recordings, mixed audio tracks where background farm noise drowns out the speaker, or unstable camera work) create noisy data that degrades machine perception. To ensure consistent input for analysis, we propose prioritizing Signal Isolation (i.e., recording speakers on separate audio tracks from environmental noise) rather than simple bitrate targets. Similarly, for field demonstrations, Visual Stability (e.g., tripod-fixed framing) is more critical than high-resolution, as camera shake introduces noise that impedes computer vision algorithms. These practices shift capture priorities from human playback quality to computational reliability, improving downstream transcription and visual analysis.

### Time-aligned visuals

A critical failure mode is the context gap regarding visual materials: speakers often reference slides or demonstrations (e.g., “as seen in this plot”) without verbalizing the data trends. In standard recordings, this visual context is lost to the AI, further constraining machine readability (Suh et al., 2024). To bridge the context gap, publishers should require Time-Aligned Slide Synchronization for presentations. Recent research (Holmberg, 2025) in automated lecture synthesis demonstrates that pure video analysis often fails to link spoken concepts to visual evidence; however, embedding the original slide deck (PDF) with timestamps allows AI agents to deterministically correlate data visuals with the spoken audio. This approach solves the unverbalized context problem without labor-intensive manual descriptions, significantly outperforming image-based matching algorithms which degrade under the variable lighting and perspective shifts common in conference recordings.

### Metadata and domain-aware curation

Audio and video content also often includes highly technical and domain-specific terminology (e.g., ‘enteric methane mitigation’) that is difficult for automated transcribers to capture accurately. As a result, audio and video materials that are informative to human experts often remain poorly interpretable by AI systems without additional structure and validation. Recent agricultural AI initiatives demonstrate both the consequences of inadequate standardization and the benefits of structured approaches. For example, Digital Green’s Farmer Chat system successfully processes YouTube extension videos through automated transcriptions and metadata tagging to create an AI-powered assistant (Singh et al., 2024). However, this system required substantial investment in custom content processing pipelines because the source videos lacked standardized transcripts and structured metadata (Singh et al., 2024). Similarly, systematic surveys of computer vision datasets for precision livestock farming have identified that the absence of contextual metadata and inconsistent annotation practices across video datasets create bottlenecks for algorithm development and evaluation (Waltz et al., 2024; Bhujel et al., 2025). Together, these examples illustrate that the lack of standardized metadata shifts the burden of interpretation onto resource-intensive pipelines rather than enabling scalable reuse.

Minimum metadata requirements should include standard bibliographic fields (title, author, curation, language) alongside AI-generated, human-verified transcripts. Such metadata provides essential contextual grounding, enabling both accurate transcription and reliable downstream reuse across datasets and platforms. We also recommend that agricultural research materials follow FAIR principles with a focus on multimodal ingestibility (Table 1). Research institutions should establish standardized repositories that support AI-assisted submission workflows (e.g., pipelines that automatically generate draft transcripts and metadata tags for authors to review)(Jones-Garcia, 2022). Governments and NGOs can further advance this goal by funding open, FAIR-compliant infrastructure, ensuring that agricultural multimedia is not just viewable by humans, but fully computable by the next generation of AI systems.

## Databases, model repositories, and scientific wikis

As scientific information shifts from static publications to dynamic webpages, agricultural research outputs (e.g., data and models) are increasingly hosted across three primary platforms: databases, model repositories, and scientific wikis. While these platforms often overlap, they present distinct challenges for AI interoperability. Diagnosing these architectural barriers is essential for transforming agricultural knowledge from human-readable web pages into agent-actionable intelligence.

### Infrastructure

The primary structural barrier is that most agricultural databases are designed for human browsing (HTML-first) rather than machine querying (API-first). For instance, FoodRisk.org provides valuable textual summaries and even allows users to execute certain models through interactive, browser-based steps. However, because these capabilities are designed primarily for human navigation rather than structured programmatic access, an AI agent cannot reliably discover which pages represent executable models, identify the required inputs and outputs in a consistent way, or invoke the model without brittle, page-specific automation.

In addition to human friendliness, it is recommended that websites are machine ready at the same time (a task increasingly tractable for general-purpose AI agents), with published resources in rigorous, machine-readable schemas, as demonstrated by the RAKIP (Risk Assessment Knowledge Integration Platform) model repository (Table 1). By enforcing the FSKX (Food Safety Knowledge Exchange) standard (Filter et al., 2024), RAKIP ensures that model artifacts and parameter files are defined in a consistent, machine-readable format. This structure enables AI agents to deterministically assess whether a model fits a specific query without having to infer capabilities from unstructured narrative descriptions. In fields where such specialized model repositories are unavailable, researchers may publish model artifacts on Hugging Face, leveraging its “Model Card” infrastructure, which allows authors to define standardized tags (e.g., task: regression, pathogen: *Listeria*) that agents use to filter and select models programmatically.

### Provenance and traceability

Trustworthiness varies widely by platform type. Model repositories (e.g., GitHub) generally set the standard for provenance: strict version control ensures every change is timestamped, attributed, and reversible, allowing AI systems to trace the exact evolution of a model. Any growing agricultural databases are encouraged to consider this traceability by implementing clear documentation of data lineage. Scientific wikis (e.g., https://www.foodrisk.org/) sit in the middle; while they provide valuable structured knowledge (e.g., parsed formulas), their edit histories are often too coarse for automated validation. Strengthening provenance requires adopting Immutable Logs and Persistent Identifiers (e.g., specific DOIs for dataset versions), enabling AI agents to verify not just what the data is, but where it came from and who certified it.

### Standardized connectivity

A persistent barrier is accessibility. Many platforms lack structured APIs, forcing researchers to rely on web scraping, which is legally precarious, computationally fragile, and prone to breaking whenever a website’s layout changes. Positive exceptions exist: OpenFDA provides the FoodAPI (openFDA, n.d.), offering a standardized, stable endpoint for querying recall data. Advancing this further, the RAKIP model repository offers an API that not only retrieves files but executes them on a containerized service, allowing AI agents to run models dynamically without local installation (Table 1).

However, such tools remain rare; to bridge this gap for resources lacking native APIs, we recommend that researchers host models as containerized Gradio or Streamlit “Spaces” on Hugging Face. By doing so, authors can leverage Hugging Face’s underlying infrastructure to automatically expose their models via predictable API endpoints. This approach transforms a standalone script into an agent-actionable service, allowing AI agents to interact with the model directly rather than requiring a manual download-and-install process.

Beyond simple connectivity, we recommend researchers abandon all-in-one code scripts in favor of “Agent Skills” (Agent Skills, n.d.), which are modular, self-contained knowledge packages that bundle executable tools (code) with semantic instructions (prompts). Unlike a simple function, a Skill explicitly teaches the agent how to perform a specific task (e.g., “conduct a *Salmonella* risk assessment”) by defining the necessary tools, data requirements, and Standard Operating Procedures (SOPs). Early agricultural systems are already adopting this pattern. NutriCHAT (Mandiga et al., 2026), a poultry nutrition agent built around four expert-curated knowledge tools, achieves 95.7% numeric accuracy on reference-grounded queries compared to 39.1% for an unaided GPT-4o baseline, while reducing hallucination by 61.9%. Similarly, knowledge-graph-grounded question answering for crop pest and disease management raises accuracy from 72.6 to 89.4% over naive RAG (Wu et al., 2026). Across both systems, performance improvements were largest for data-intensive retrieval tasks requiring precise numerical knowledge, suggesting that performance scales with the quality and structure of the underlying knowledge base. NutriCHAT’s results further show that improvements narrow for open-ended queries where general-purpose models remain competitive. To make these improvements transparent and reproducible, these skills should output runtime provenance logs (i.e., a machine-readable chain of thought detailing exactly which parameters and data versions were used; Table 1).

For high-stakes scientific reasoning where precise execution is required, these skills should be implemented via the Model Context Protocol (MCP) (Model Context Protocol, n.d.). Rather than relying on natural language prompting alone, MCP acts as a standardized connector that allows platforms to expose their internal data and models as strictly defined resources and tools. Therefore, we recommend a two-stage evolution: first, agricultural platforms must expose their data via Standardized APIs (e.g., OpenAPI specs); second, they should wrap these APIs in MCP servers (open-sourced agent skills such as mcp-builder can facilitate this development). This ensures that agents can securely query databases, execute models, and extract wiki knowledge through stable, authorized functions rather than fragile, hand-written scrapers.

## Discussion

The transition to agentic AI demands that agricultural research evolve from a collection of static, human-readable documents into a network of dynamic, computable assets. This requires a paradigm shift where agent-actionability becomes as critical as scientific rigor, ensuring that all text, dataset, model, and multimedia resources are structurally prepared for autonomous reasoning. Achieving this shift, however, requires honest acknowledgment of the tradeoffs it introduces.

### Tensions and considerations

These principles are not free of tradeoffs. First, the capacity to produce, host, and access agent-actionable infrastructure is distributed unequally, weighing most on smaller research groups and institutions in low-resource settings, an equity concern that has long shadowed FAIR and FAIR4RS adoption (Bezuidenhout et al., 2017; Shanahan and Bezuidenhout, 2022). Compute disparities translate directly into research throughput, with iteration cycles running multi-day in low-resource settings versus minutes in well-resourced laboratories (Channing and Ghosh, 2026). Many institutions in these settings lack the technical capacity to host APIs, maintain MCP servers, or curate machine-readable metadata, barriers that infrastructure alone cannot address. Second, building scientific workflows atop frontier LLMs and their associated protocols (e.g., MCP) concentrates dependence on a small set of proprietary providers, creating fragility against future pricing, policy, or capability shifts. Third, agent-actionability accelerates downstream errors as readily as correct inferences: an agent that confidently chains together poorly curated sources can amplify hallucinations into operational decisions, particularly consequential in food safety where errors may lead to public health impact. Such failures are already documented beyond agriculture: leading models elaborated on fabricated clinical details inserted into decision-support prompts in 50–82% of cases (Omar et al., 2025), with comparably high hallucination rates reported for legal queries (Dahl et al., 2024). Fourth, agent-mediated reuse raises unresolved questions of provenance and credit: current norms specify neither when outputs may be ingested by agents, nor how the data curators and infrastructure builders behind them should be credited when agents act on their work (Channing and Ghosh, 2026). Addressing these questions will require concrete governance mechanisms, including machine-readable consent frameworks that specify agent usage permissions, attribution standards for automated reuse, and institutional policies for human oversight in high-stakes applications. We therefore view FAIR4AG^2^ as necessary but not sufficient. Realizing its benefits will require parallel investment in equitable participation, careful curation, human-in-the-loop verification for high-stakes decisions, open standards that resist vendor lock-in, and governance norms that credit curators and infrastructure builders.

### Path forward

This vision is no longer theoretical, but a strategic imperative actively supported by federal priorities, as evidenced by the USDA NIFA-funded *Terrascope* initiative (Open Data Framework|NIFA, n.d.), which is operationalizing these principles to build AI-powered open data frameworks. To sustain and scale this momentum, we call on funding agencies to mandate “Data Stewardship” budget lines in grant proposals, ensuring resources are allocated not just for novelty, but for the curation required to make outputs agent-ready. In parallel, the agricultural science community must prioritize the creation of “Gold Standard” benchmark datasets (i.e., verified, high-quality ground truth for key agricultural tasks) to reliably train and validate emerging scientific agents. As a perspective, this work is conceptual rather than empirical. Systematic benchmarking of agricultural resources against these principles, together with development of the maturity criteria in Table 1 into a validated assessment instrument, remains an important direction for future work, itself dependent on the gold-standard datasets described above. By committing to these standards of interoperability and transparency, the community ensures that today’s scientific outputs do not become tomorrow’s “dark data,” but rather the foundational logic for the next generation of AI-driven agricultural solutions.

## Data Availability

The original contributions presented in the study are included in the article/supplementary material, further inquiries can be directed to the corresponding author.

## References

[ref1] Agent Skills (n.d.). Available online at: https://developers.openai.com/codex/skills (Accessed February 11, 2026).

[ref2] AGROVOC. (n.d.). Multilingual thesaurus—AGROVOC. Available online at: https://www.overleaf.com/about/ai-features (Accessed January 22, 2026).

[ref3] AI features—Overleaf, Online LaTeX Editor. (n.d.). Available online at: https://www.overleaf.com/about/ai-features?gad_source=1&gad_campaignid=22787343279&gbraid=0AAAAAqfJXW6IrtqcEDKSfNA7JoZfl6eGw&gclid=CjwKCAiAssfLBhBDEiwAcLpwfjybMAUFiIvUlFrgJvzdiMkx6UCe9oTwk1PM-fVEacK-CBwyN1vnCRoCQxgQAvD_BwE (Accessed January 21, 2026).

[ref4] Awais M. Salem Abdulla Alharthi A. H. Kumar A. Cholakkal H. Anwer R. M. (2025). AgroGPT: efficient agricultural vision-language model with expert tuning. Proceedings—2025 IEEE Winter Conference on Applications of Computer Vision, WACV 2025. 5687–5696.

[ref5] Barker M. Chue Hong N. P. Katz D. S. Lamprecht A. L. Martinez-Ortiz C. Psomopoulos F. . (2022). Introducing the FAIR principles for research software. Sci. Data 9:622. doi: 10.1038/s41597-022-01710-x 36241754 PMC9562067

[ref6] Bezuidenhout L. M. Leonelli S. Kelly A. H. Rappert B. (2017). Beyond the digital divide: towards a situated approach to open data. Sci. Public Policy 44, 464–475. doi: 10.1093/SCIPOL/SCW036

[ref7] Bhujel A. Wang Y. Lu Y. Morris D. Dangol M. (2025). A systematic survey of public computer vision datasets for precision livestock farming. Comput. Electron. Agric. 229:109718. doi: 10.1016/J.COMPAG.2024.109718

[ref9001] Cabrera V. E. Bewley J. Breunig M. Breunig T. Cooley W. De Vries A. . (2025). Data integration and analytics in the dairy industry: challenges and pathways forward. Animals. 15:329. doi: 10.3390/ani15030329 39943099 PMC11816260

[ref8] Channing G. Ghosh A. (2026). AI for scientific discovery is a social problem. Patterns 7:101497. doi: 10.1016/J.PATTER.2026.101497, 42028402 PMC13100680

[ref9] Dahl M. Magesh V. Suzgun M. Ho D. E. (2024). Large legal fictions: profiling legal hallucinations in large language models. J. Legal Anal. 16, 64–93. doi: 10.1093/JLA/LAAE003

[ref10] Filter M. Schüler T. Ben Romdhane R. (2024). Food Safety Knowledge Exchange (FSKX) format: current status and strategic development plans based on a SWOT analysis. Microb. Risk Anal. 27:100309. doi: 10.1016/J.MRAN.2024.100309

[ref11] FoodOn. (n.d.) FoodOn: a farm to fork ontology. Available online at: https://foodon.org/ (accessed February 11, 2026).

[ref12] Gontijo D. Rolins Santana D. de Assis Costa G. Cabrera V. E. Noronha de Andrade Freitas E. (2025). Dairy GPT: empowering dairy farmers to interact with numerical databases through natural language conversations. Smart Agric. Technol. 12:101097. doi: 10.1016/J.ATECH.2025.101097

[ref13] Holmberg A. (2025). Generating narrated lecture videos from slides with synchronized highlights. Proceedings of the 34th ACM International Conference on Information and Knowledge Management (CIKM ‘25) 1

[ref14] Jones-Garcia E. (2022). Speech recognition, machine translation, and corpus analysis for identifying farmer demands and targeting digital extension. Available online at: https://hdl.handle.net/10568/127005 (Accessed January 22, 2026).

[ref15] Jonquet C. Toulet A. Arnaud E. Aubin S. Dzalé Yeumo E. Emonet V. . (2018). AgroPortal: a vocabulary and ontology repository for agronomy. Comput. Electron. Agric. 144, 126–143. doi: 10.1016/j.compag.2017.10.012

[ref16] Kocetkov D. Li R. Allal L. B. Li J. Mou C. Ferrandis C. M. . (2022). The stack: 3 TB of permissively licensed source code. Trans. Mach. Learn. Res. 2023. Available online at: https://arxiv.org/pdf/2211.15533 (Accessed July 15, 2026) preprint.

[ref17] Kpodo J. Nejadhashemi A. P. (2025). Navigating challenges/opportunities in developing smart agricultural extension platforms: multi-media data mining techniques. Artif. Intell. Agric. 15, 426–448. doi: 10.1016/J.AIIA.2025.04.001

[ref18] LandingAI: Computer Vision Platform—Visual AI Software. (n.d.). Available online at: https://landing.ai/ (Accessed January 21, 2026).

[ref19] Liu E. Yang H. Sharma S. van Leerdam M. B. Niu P. VandeHaar M. J. . (2025). Agents are all you need: pioneering the use of agentic artificial intelligence to embrace large language models into dairy science. J. Dairy Sci. 108, 14038–14049. doi: 10.3168/JDS.2025-26775, 40945783

[ref20] Longpre S. Mahari R. Chen A. Obeng-Marnu N. Sileo D. Brannon W. . (2024). A large-scale audit of dataset licensing and attribution in AI. Nat. Mach. Intell. 6, 975–987. doi: 10.1038/s42256-024-00878-8

[ref21] Mandiga A. Yoon J. H. Kasireddy B. Olukosi O. A. Li G. (2026). NutriCHAT: a reasoning-driven large language model agent with expert-designed tools for knowledge-grounded poultry nutrition assistance. Comput. Electron. Agric. 245:111564. doi: 10.1016/J.COMPAG.2026.111564

[ref22] Model Context Protocol. (n.d.) What is the Model Context Protocol (MCP)? Available online at: https://modelcontextprotocol.io/docs/getting-started/intro (Accessed December 4, 2025).

[ref23] olmOCR—Open-Source OCR for Accurate Document Conversion. (n.d.). Available online at: https://playground.allenai.org/model/olmocr-2-7b-1025 (Accessed January 21, 2026).

[ref24] Omar M. Sorin V. Collins J. D. Reich D. Freeman R. Gavin N. . (2025). Multi-model assurance analysis showing large language models are highly vulnerable to adversarial hallucination attacks during clinical decision support. Commun. Med. 5:330. doi: 10.1038/S43856-025-01021-3, 40753316 PMC12318031

[ref25] Open Data Framework|NIFA. (n.d.). Available online at: https://www.nifa.usda.gov/grants/funding-opportunities/open-data-framework (Accessed February 11, 2026).

[ref26] openFDA. (n.d.). Available online at: https://open.fda.gov/apis/food/ (Accessed December 4, 2025).

[ref27] Paperclip. (n.d.). Available online at: https://paperclip.gxl.ai/ (Accessed May 8, 2026).

[ref28] Prism|A free, LaTeX-native workspace for scientists|OpenAI. (n.d.). Available online at: https://openai.com/prism/ (Accessed February 11, 2026).

[ref29] Shanahan H. Bezuidenhout L. (2022). Rethinking the a in FAIR data: issues of data access and accessibility in research. Front. Res. Metr. Anal. 7:912456. doi: 10.3389/frma.2022.912456 35965666 PMC9363602

[ref30] Singh N. Wang’ombe J. Okanga N. Zelenska T. Repishti J. Jayasankar G. K. . (2024). Farmer.Chat: scaling AI-powered agricultural services for smallholder farmers. Proceedings of (Conference acronym ‘XX) 1. Available online at: https://arxiv.org/pdf/2409.08916 (Accessed January 22, 2026).

[ref31] Suh J. Na I. Jung W. (2024). Improving domain-specific ASR with LLM-generated contextual descriptions. Available online at: https://arxiv.org/pdf/2407.17874 (Accessed January 22, 2026).

[ref32] Swanson K. Wu W. Bulaong N. L. Pak J. E. Zou J. (2025). The virtual lab of AI agents designs new SARS-CoV-2 nanobodies. Nature 646, 716–723. doi: 10.1038/s41586-025-09442-9, 40730228

[ref33] Top J. Janssen S. Boogaard H. Knapen R. Şimşek-Şenel G. (2022). Cultivating FAIR principles for agri-food data. Comput. Electron. Agric. 196:106909. doi: 10.1016/j.compag.2022.106909

[ref34] Waltz L. Katari S. Hong C. Anup A. Colbert J. Potlapally A. . (2024). Cyberinfrastructure for machine learning applications in agriculture: experiences, analysis, and vision. Front. Artif. Intell. 7:1496066. doi: 10.3389/frai.2024.1496066 39917547 PMC11798914

[ref35] Wilkinson M. D. Dumontier M. Aalbersberg I. J. Appleton G. Axton M. Baak A. . (2016). The FAIR guiding principles for scientific data management and stewardship. Sci. Data 3:160018. doi: 10.1038/sdata.2016.18 26978244 PMC4792175

[ref36] Wu H. Xie N. Wang X. Fan J. Li Y. Meng Z. (2026). Crop GraphRAG: pest and disease knowledge base Q&A system for sustainable crop protection. Front. Plant Sci. 16:1696872. doi: 10.3389/fpls.2025.1696872 41560917 PMC12812888

